# Neurological and Functional Outcomes After Decompression With or Without Fusion for Thoracic Ossification of the Ligamentum Flavum: A Systematic Review and Evidence Synthesis

**DOI:** 10.7759/cureus.116563

**Published:** 2026-09-19

**Authors:** Ajaybir Singh, Amanpreet Singh, Supreet Kaur, Jagdeep Singh, Kapil Bansal

**Affiliations:** 1 Department of Orthopaedics, Guru Gobind Singh Medical College and Hospital, Faridkot, IND; 2 Department of Pathology, District Hospital, Faridkot, IND

**Keywords:** decompressive laminectomy, ligamentum flavum calcification, ossification, spinal-fusion, thoracic spine surgery

## Abstract

Thoracic ossification of the ligamentum flavum (TOLF) is an important cause of thoracic myelopathy. This systematic review evaluated neurological and functional outcomes after posterior decompression with or without fusion for TOLF and assessed perioperative safety.

An electronic search of PubMed/MEDLINE was conducted according to the Preferred Reporting Items for Systematic Reviews and Meta-Analyses 2020 guidelines. Studies published up to December 2024 that reported adult patients undergoing posterior decompression with or without fusion for TOLF were included. Neurological recovery was assessed using the modified Japanese Orthopaedic Association score and recovery rate. Perioperative outcomes included cerebrospinal fluid leakage, blood loss, infection, and operative time. Owing to clinical and methodological heterogeneity, a narrative synthesis was performed using the Synthesis Without Meta-analysis framework. Risk of bias was assessed using ROBINS-I, and the certainty of evidence was evaluated using the GRADE approach.

Ten retrospective cohort studies including 846 patients were included. Regarding the primary neurological outcomes and perioperative safety, the use of an ultrasonic osteotome reduced operative time, blood loss, and dural tear rates compared with a high-speed burr, without significant differences in long-term neurological outcomes. Dural ossification was the strongest predictor of cerebrospinal fluid leakage. Regarding secondary prognostic factors, longer symptom duration, preoperative intramedullary T2 hyperintensity, and lower baseline modified Japanese Orthopaedic Association scores were associated with poorer neurological recovery. Furthermore, a signal change ratio ≥1.54 on quantitative magnetic resonance imaging and sagittal beak-type ossification were associated with unfavorable prognosis. Overall certainty of evidence was low to very low.

Outcomes after surgery for TOLF are primarily determined by preoperative neurological status and magnetic resonance imaging features. These findings may help guide surgical timing and selective fusion, while operative technique appears to mainly influence perioperative safety.

## Introduction and background

Ossification of the ligamentum flavum (OLF) is a pathological process in which the elastic fibers of the ligamentum flavum are progressively replaced by mature lamellar bone through endochondral ossification [1]. This is not a passive calcification process but an active, biologically regulated form of ectopic bone formation in which the ligament hypertrophies and ossifies, leading to dorsal compression of the spinal cord [2]. 

Epidemiologically, OLF shows a marked predilection for East Asian populations, while it is relatively uncommon in Caucasian populations, with imaging-based prevalence estimates ranging from 0.1% to 1.3% [3,4]. Anatomically, thoracic ossification of the ligamentum flavum (TOLF) shows a bimodal distribution with a marked predilection for the lower thoracic spine. The T10-T11 segment is the most frequently affected (44.0%), followed closely by T11-T12 (41.6%) [4]. 

The etiology of OLF is multifactorial and likely reflects an interaction between genetic predisposition, biomechanical stress, and metabolic factors. At the molecular level, osteogenic and angiogenic mediators, including RUNX2, vascular endothelial growth factor (VEGF), transforming growth factor (TGF-β), and bone morphogenetic protein-2 (BMP-2), have been implicated in the development of ectopic bone formation within the ligamentum flavum [5,6]. Genetic susceptibility may also contribute, although evidence regarding specific variants such as COL6A1 remains inconsistent [7,8]. Experimental studies further suggest that mechanical loading can promote osteogenic differentiation of ligamentum flavum cells through pathways involving RUNX2 and BMP-2 [9]. Collectively, these findings support the concept that OLF is an active and progressive ossification process rather than simple passive calcification, resulting in spinal cord compression, neurological impairment, and associated imaging characteristics.

Dural ossification (DO) is identified on axial CT by characteristic signs such as the “tram-track” and “comma” signs [10]. OLF is commonly described using classifications proposed by Sato et al. (lateral, extensive, enlarged, fusion, nodular types) based on computed tomography (CT) findings and by Kuh et al. (beak and round types) based on magnetic resonance imaging (MRI) [11,12].

The typical initial symptoms of myelopathy due to TOLF include numbness, paresthesia in the lower extremities, and gait instability. With progressive compression, patients develop spastic paraparesis, increased muscle tone, and eventually bladder or bowel dysfunction [2]. OLF at the T12-L1 level involving the conus medullaris often presents a mixed picture of upper motor neuron (UMN) and lower motor neuron (LMN) signs, frequently accompanied by early sphincter dysfunction. An early surgical intervention is recommended for TOLF patients presenting with spastic gait, marked loss of muscle power in lower extremities, or bladder and bowel disturbance [2]. Enblock laminectomy or posterior decompression alone (D) remains the most commonly performed procedure, but the role of adding instrumented fusion (DF) continues to be debated. Neurological outcomes, prognostic factors, and complications following surgery for TOLF are an active area of research interest, given the progressive nature of disease and multiple factors influencing the quality of life. 

In this systematic review, we aimed primarily to determine the neurological and functional outcomes after posterior laminectomy with or without fusion. Secondary objectives were to assess the perioperative safety of the surgical procedure, identify risk factors influencing the functional outcome, and analyze complications associated with surgery for TOLF.

Methods

Protocol and Registration

The review was conducted in accordance with the Preferred Reporting Items for Systematic Reviews and Meta-Analyses (PRISMA) 2020 guidelines [13] and followed the PICO framework. The protocol was registered in the PROSPERO database (CRD420251173526).

Literature Search Strategy 

A systematic search of PubMed/MEDLINE was performed to identify studies published during the past 10 years, with the final search executed on December 31, 2024. The PubMed "English language" filter was applied. Search terms included relevant keywords from titles and abstracts as well as medical subject headings (MeSH). The keywords used for the search, along with Boolean combinations, included decompression, laminectomy, ligamentum flavum calcification, ossification, spinal fusion, and thoracic spine surgery. To maximize sensitivity, a multistep search strategy was employed rather than a single Boolean string, utilizing various combinations of keywords and MeSH terms across multiple discrete searches. Reference lists of eligible articles and relevant reviews were also screened to identify additional studies. Two reviewers independently screened titles and abstracts, followed by full-text assessment of potentially eligible articles. Disagreements were resolved through discussion and, when required, consultation with a third reviewer. The study selection process is presented in the PRISMA flow diagram (Figure 1).

**Figure 1:**
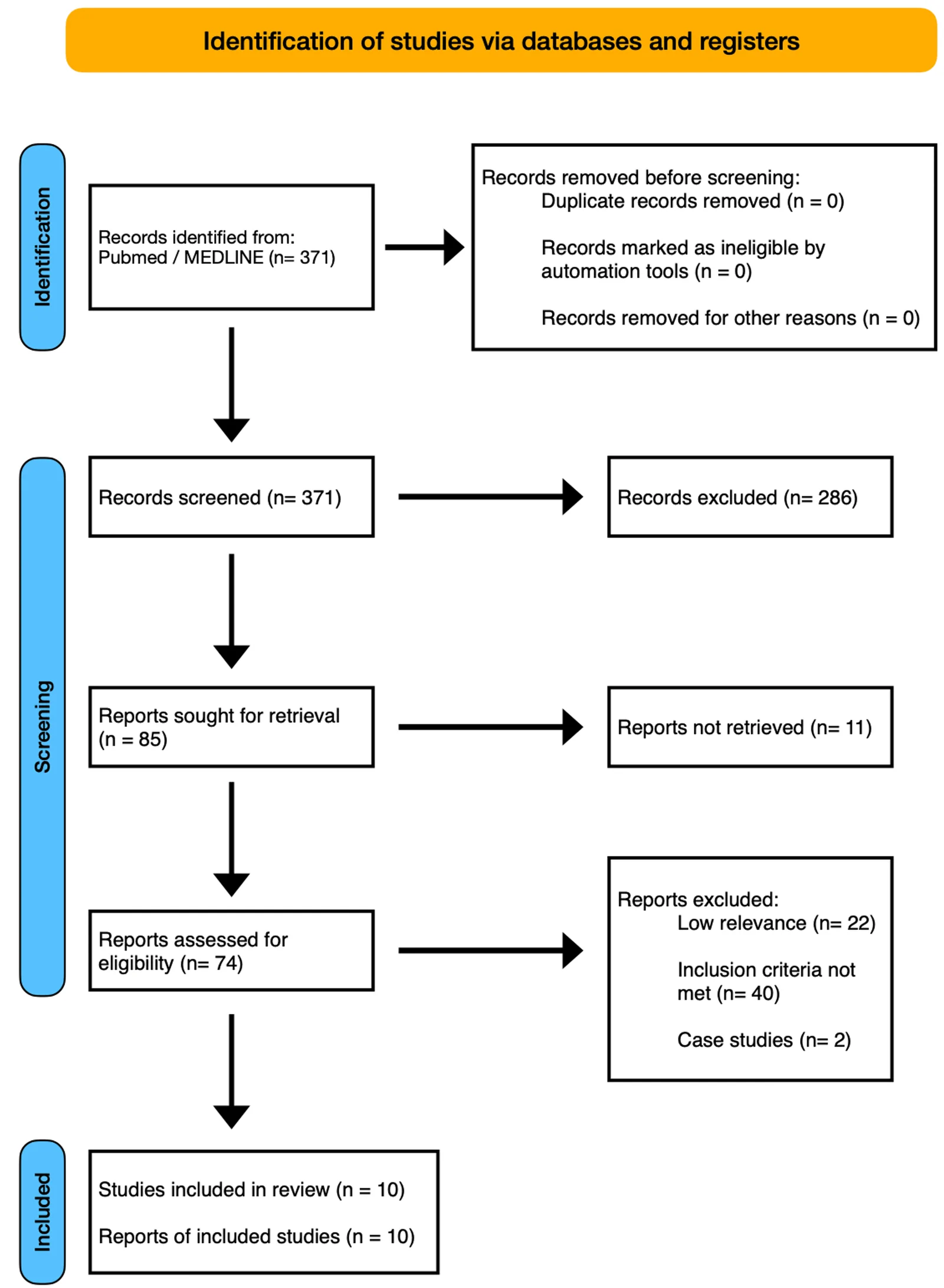
PRISMA 2020 flow diagram for new systematic reviews, which included searches of databases and registers only Ref. [13].

Eligibility criteria 

Titles and abstracts were screened according to predefined inclusion criteria. Eligible studies were full-text articles in English involving human subjects and included randomized controlled trials, case series, and retrospective or prospective observational and cohort studies.

Population: Studies were required to include at least 30 adult patients (≥18 years) with TOLF diagnosed on MRI or CT and presenting with clinical features of myelopathy.

Intervention: Surgical interventions included posterior decompression using laminectomy, en bloc laminectomy (D), or laminectomy with posterior instrumented fusion (DF).

Control: A control group was not mandatory.

Outcomes: Patient-reported primary outcomes were neurological recovery measured using the modified Japanese Orthopaedic Association score (mJOA) and Hirabayashi recovery rate (RR) [14,15]. Secondary outcomes included perioperative complications such as cerebrospinal fluid (CSF) leakage, blood loss, operative time, and new neurological deficits. Only studies with a minimum follow-up of two years were included. 

Studies involving endoscopic procedures, laminoplasty, alternative decompression techniques, tandem stenosis (coexisting symptomatic cervical or lumbar canal stenosis along with TOLF), or non-OLF causes of thoracic myelopathy were excluded.

Data Extraction and Quality Assessment 

Ten studies met the inclusion criteria after completion of the PRISMA selection process. The data were extracted from each study using a structured table that included information on study design, sample size, follow-up duration, intervention or study arm details, comparison groups (if any), neurological outcomes, and complications. Because of heterogeneity in surgical techniques, patient characteristics, and outcome reporting, results were synthesized using a structured narrative approach in accordance with the Synthesis Without Meta-analysis (SWiM) guideline [16]. Two reviewers independently assessed risk of bias for all included studies using the ROBINS-I tool across seven domains [17]. Certainty of evidence was evaluated using the Grading of Recommendations Assessment, Development, and Evaluation (GRADE) framework [18].

Synthesis Methods

To ensure transparency in this qualitative synthesis, the following explicit synthesis rules for SWiM were applied:

Rule 1 (Grouping): Studies were grouped by outcome domain (neurological recovery, surgical safety, and complications) and intervention type (surgical tools and instrumentation status)

Rule 2 (Standardization): Neurological outcomes were standardized using the Hirabayashi RR and mJOA scores.

Rule 3 (Synthesis method): Synthesis was performed by comparing the direction and significance of effects across studies (vote counting based on p-values) and identifying consistent prognostic thresholds.

Rule 4 (Prioritization): Priority was given to multicenter studies (e.g., Li et al. [19], Zhang et al. [20]) and studies with larger cohorts (e.g., Hu et al. [21], Wang et al. [22]) to minimize single-center bias.

Rule 5 (Certainty assessment): Evidence certainty was assessed using the GRADE framework, acknowledging that retrospective cohort designs start at a "Low" certainty level. 

## Review

Results

Study Characteristics

The study selection process is illustrated in the PRISMA flow diagram [13] (Figure 1), which led to the inclusion of 10 studies. The main characteristics of these studies are presented in Table 1, while their reported outcomes are summarized in Table 2.

**Table 1:** Characteristics of the studies included in the systematic review SD = standard deviation; L = laminectomy; LF = laminectomy with instrumented fusion; HSB = high-speed burr; UO = ultrasonic osteotome; R = retrospective study; RM = retrospective multicenter study. *Study by Liu et al. (2022) [28] does not report the specific mean follow-up duration. However, minimum follow-up of 24 months was a mandatory inclusion criterion in the study for all patients.

S. No.	Author (year)	Publication journal	Country	Study type	No. of patients (N)	Mean ± SD follow-up (months)	Procedure	Level of evidence
1	Zhang et al. (2023) [20]	Global Spine J	China	RM	94	34.9 ± 19.5	L	Level III
2	Zhang et al. (2016) [23]	Sci Rep	China	R	96	62.4	L = 86, LF = 10	Level III
3	Sun et al. (2022) [24]	Med Sci Monit	China	R	64	31.85 ± 7.66	L = 44, LF = 20	Level III
4	Li et al. (2016) [19]	Spinal Cord	China	RM	85	49.2	L = 57, LF = 28	Level III
5	Du et al. (2022) [25]	J Orthop Surg Res	China	R	48	27.2 ± 5.3	LF	Level III
6	Wang et al. (2021) [26]	Pain Res Manag	China	R	57	34	L = 40, LF = 17	Level III
7	Bagga et al. (2023) [27]	Global Spine J	India	R	77	60.8 ± 26.0	LF (HSB vs UO)	Level III
8	Liu et al. (2022) [28]	Front Surg	China	R	32	24*	LF	Level III
9	Hu et al. (2023) [21]	Orthop Surg	China	R	151	83.1 ± 18.5	En block LF	Level III
10	Wang et al. (2021) [22]	BMC Musculoskelet Disord	China	R	142	29.6 ± 12.4	L = 77, LF = 65	Level III

**Table 2:** Summary of clinical outcomes and prognostic factors in thoracic OLF surgery N = number of patients; mJOA = modified Japanese Orthopaedic Association score; RR = recovery rate; UO = ultrasonic osteotome; HSB = high-speed burr; LI = laminectomy with instrumentation; LA = laminectomy alone; DO = dural ossification; SCR = signal change ratio; ISI = increased signal intensity; CSF = cerebrospinal fluid; OLF = ossified ligamentum flavum; SCR = signal change ratio.

S. no.	Study (author, year)	Neurological recovery mJOA (preoperative/postoperative), RR	Key prognostic findings and complications	CSF leaks (N)
1	Zhang et al. (2023) [20]	mJOA = 7.3/9.1; RR = 50.2%	ISI Grade: Grade 2 (intense) ISI associated with worst RR (35.8%) and highest DO (91.3%) and CSF leak (65.2%) rates.	Total CSF leak = 27. In Grade 0. ISI = 2. In Grade 1, ISI = 10. In Grade 2, ISI = 15
2	Zhang et al. (2016) [23]	mJOA = 5.5/8.0; RR = 45.8%	Preoperative SCR ≥1.54 (OR 2.86, 95% CI: 1.17-6.97, p = 0.021) and symptom duration >12 months significantly predicted poor outcomes.	Total CSF leak = 7. Good recovery group = 3. Poor recovery group = 4
3	Sun et al. (2022) [24]	mJOA = 5.03/8.11; RR = 53.04%	Risk factors (multivariate): Symptom duration >17 months (OR 1.08, 95% CI: 1.01-1.15, p = 0.022), intramedullary high signal intensity (OR 16.02, 95% CI: 1.95-131.59, p = 0.010), dural ossification (OR 14.00, 95% CI: 2.07-94.83, p = 0.007), and beak-type morphology (OR 4.72, 95% CI: 1.17-19.09, p = 0.029).	CSF leak cases = NA. Cases with dural ossification = 14
4	Li et al. (2016) [19]	mJOA = 3.8/8.2; RR = 63%	Predictors: Middle thoracic level, symptom duration, T2 signal change, and baseline severity of myelopathy predicted poor outcomes.	Total dural tear = 17. Total CSF leak = 9 (7 cases with DO)
5	Du et al. (2022) [25]	mJOA (beak) = 6.03/7.74; mJOA (round) = 6.95/8.93	Morphology: Beak-type morphology associated with significantly lower RR (38.3%) vs. round-type (53.2%)	CSF leak = 5
6	Wang et al. (2021) [26]	RR = 54.8%	Dissatisfaction risk: Linked to diabetes; prolonged duration of symptoms; high expenses in short term; low RR and recurrence of symptoms in long term.	Total CSF leak = 8. Satisfied group = 2. Dissatisfied group = 6
7	Bagga et al. (2023) [27]	mJOA = 4.3/7.6; RR: 49.9%	Predictors: Symptom duration, cord signal change, and preoperative severity. UO significantly reduced dural tears (UO 12.5% vs HSB 29%).	Dural tear = 17. In HSB group = 13. In UO group = 4
8	Liu et al. (2022) [28]	RR = 50.8% (DO); RR = 53.2% (non-DO)	DO impact: DO led to longer surgery and 100% CSF leak rate in the DO cohort, but similar neurological recovery compared to non-DO group.	DO group = 12. Non-DO group = 0
9	Hu et al. (2023) [21]	mJOA (both groups) = 5/10; RR (early) = 80%; RR (late group) = 81%	Safety: Learning curve asymptote at 76 cases; experience reduced dural tears (31.6% to 17.3%) but did not affect RR.	CSF leak = 49. Early group = 29. Late group = 20
10	Wang et al. (2021) [22]	RR (LA) = 38.3%; RR (LI) = 46.9%	Benefit: LI showed superior outcomes in severe cases (canal area <60%), beak-type OLF, and longer symptom duration.	LA group = 4. LI group = 22

A structured narrative synthesis was performed for the 10 included studies using the SWiM framework. Although outcome measures were largely comparable across studies, a meta-analysis was not feasible due to heterogeneity in surgical techniques and patient populations.

MRI Signal Intensity

Preoperative intramedullary signal alterations on T2-weighted MRI demonstrated a strong association with baseline neurological status and postoperative outcomes. In a multicenter study of 94 patients, Zhang et al. categorized cord signal changes as Grade 0 (absent), Grade 1 (mild), and Grade 2 (marked). Patients without signal changes (Grade 0) presented with less severe myelopathy and higher preoperative JOA scores, whereas those with Grade 2 changes experienced inferior neurological recovery, longer operative duration, increased blood loss, and higher complication rates [20].

Using a quantitative approach, Zhang et al. further evaluated the signal change ratio (SCR) and identified preoperative SCR ≥1.54 as an independent predictor of poor surgical outcome on multivariate logistic regression (OR 2.86, 95% CI: 1.17-6.97, p = 0.021) [23]. Consistent findings were reported by Sun et al., who demonstrated that intramedullary T2-weighted hyperintensity independently predicted reduced postoperative neurological recovery [24]. Similarly, in a cohort of 85 patients, Li et al. showed through subgroup analysis that the presence of T2-weighted intramedullary signal change was a significant negative prognostic factor, likely reflecting irreversible spinal cord pathology [19].

SWiM conclusion: Consistent evidence (four of four studies reporting this metric) for worse outcomes with increasing intramedullary signal change.

Morphology of Ossified Ligamentum Flavum 

The shape of the ossified ligamentum flavum influenced recovery. Du et al. reported that sagittal (beak vs round) morphology had a significant effect: patients with beak-type OLF had significantly lower postoperative JOA scores and RRs than those with round-type ossification (p = 0.010 and p = 0.034, respectively). Axial configuration (unilateral vs bilateral) had no impact on outcome [25]. Similarly, a beak (sagittal) configuration was independently associated with poor outcome in regression analysis. This finding aligns with the hypothesis that beak-shape OLF puts more pressure on the spinal cord and increases surgical difficulty [24].

SWiM conclusion: Moderate consistency (two of three studies reporting this metric) supporting beak-type morphology as an adverse prognostic factor.

Symptom Duration and Preoperative Severity

Longer preoperative symptom duration was consistently linked to worse recovery [26]. As an independent risk factor for poor surgical outcome (RR < 50%), Zhang et al. found that symptoms >12 months (vs <6 months) nearly quadrupled the odds of poor surgical outcome [23]. Sun et al. likewise identified symptom duration over 17 months as a risk factor for poor JOA improvement [24]. In linear regression analysis, studies identified that severe baseline myelopathy also correlated with outcome: patients with lower preoperative JOA scores (severe deficits) had lower RRs [19]. These findings indicate that chronic, severe cord compression leads to poor outcomes. In a multivariate linear regression analysis by Bagga et al., the most significant predictor of outcome identified was shorter symptom duration (one month), which correlated with better recovery (p = 0.0001) [27].

SWiM conclusion: Strong consistency (five of five studies reporting this metric) for symptom chronicity and baseline severity as negative prognostic factors.

Dural Ossification 

A comparative retrospective study conducted by Liu et al. on single-level TOLF revealed an incidence of DO at 37.5%. The location of DO was predominantly observed in the lower thoracic spine, with 75% of cases situated between T9 and T12. Patients in the DO group experienced significantly extended operation durations (94.75 minutes compared to 80.00 minutes), higher blood loss (331.67 milliliters compared to 253.00 milliliters), prolonged hospital stays (13.83 days compared to 10.05 days), and elevated cerebrospinal fluid (CSF) leak rates (all p < 0.001). However, neurological recovery was similar between groups, with comparable RR and health-related quality of life (HRQOL) scores (SF-36) [28]. Consistently, Sun et al. reported that DO was a primary independent risk factor for poor outcomes [24].

SWiM conclusion: Consistent association (two of two studies reporting this metric) with perioperative morbidity; inconsistent association with neurological recovery.

Surgical Technique 

Use of different surgical techniques affected perioperative outcomes. Bagga et al. performed a subgroup analysis comparing the conventional high-speed burr (HSB) technique with the use of an ultrasonic osteotome (UO). The UO group had significantly fewer dural tears (12.5% vs. 29%) and shorter operative time (mean 125.8 vs. 189.4 minutes, p < 0.001). This finding supports the growing consensus on the benefits of tissue-selective devices for minimizing mechanical injury during challenging decompressions. Despite improvements in safety and operative time, there is no statistically significant difference in long-term neurological RRs between the two techniques [27].

Hu et al. analyzed the learning curve of UO-based en bloc laminectomy for TOLF and compared surgical outcomes at different periods of surgeon experience. The operative time and blood loss decreased significantly with experience. The learning curve for mastering the technique was relatively long and plateaued by 76 cases. An increase in surgeon experience reduced the dural tear rate (31.6% early vs. 17.3% later, p = 0.042) and shortened the hospital stay. Both early and late groups achieved similar median RRs (80.0% vs. 81.3%) and final mJOA scores. Hence, the surgeon’s experience primarily influenced surgical parameters and immediate safety rather than long-term functional recovery [21].

SWiM conclusion: Technique and experience affect safety, not long-term neurological recovery (two of two studies reporting this metric).

Fusion Versus Decompression Only 

Wang et al. in a comparative study of 142 patients conclude that instrumentation is superior for severe TOLF, specifically for patients with long symptom duration, intramedullary signal changes, DO, beak-type morphology, and low preoperative mJOA scores [22]. Instrumentation provides spinal stability and significantly reduces the risk of postoperative kyphosis. Thus, tailoring the surgical approach (technique and instrumentation) to disease severity can improve outcome. Conversely, Li et al. in a univariate analysis revealed that being "aided by fusion with instrumentation" was not significantly related to the final surgical RR (p = 0.1814) [19]. Similarly, Sun et al. found no statistically significant difference in the choice of surgical method between the "good prognosis" and "poor prognosis" groups (p = 0.249) [24].

SWiM conclusion: Inconsistent evidence; one of three studies found a benefit of fusion for severe cases, while two of three studies found no significant difference.

Risk of Bias

Risk of bias, assessed using the ROBINS-I tool, is detailed in Table 3.

**Table 3:** Risk-of-bias assessment of included studies using ROBINS-I D1 = bias due to confounding; D2 = selection of participants; D3 = classification of interventions; D4 = deviations from intended interventions; D5 = missing data; D6 = measurement of outcomes; D7 = selection of reported results.

Study	D1: Confounding	D2: Selection	D3: Classification	D4: Deviations	D5: Missing data	D6: Measurement	D7: Reporting	Overall risk
Zhang et al. (2023) [20]	Serious	Low	Low	Moderate	Low	Moderate	Low	Serious
Zhang et al. (2016) [23]	Serious	Moderate	Low	Moderate	Low	Moderate	Low	Serious
Sun et al. (2022) [24]	Serious	Moderate	Low	Moderate	Low	Moderate	Low	Serious
Li et al. (2016) [19]	Serious	Low	Low	Moderate	Moderate	Moderate	Low	Serious
Du et al. (2022) [25]	Serious	Moderate	Low	Moderate	Serious	Moderate	Low	Serious
Wang et al. (2021) [26]	Serious	Moderate	Low	Moderate	Low	Moderate	Low	Serious
Bagga et al. (2023) [27]	Serious	Moderate	Low	Moderate	Moderate	Moderate	Low	Serious
Liu et al. (2022) [28]	Serious	Moderate	Low	Moderate	Low	Moderate	Low	Serious
Hu et al. (2023) [21]	Serious	Low	Low	Moderate	Moderate	Moderate	Low	Serious
Wang et al. (2021) [22]	Serious	Moderate	Low	Moderate	Low	Moderate	Low	Serious

The data show bias due to confounding at serious risk (D1): all 10 studies are retrospective and non-randomized, meaning surgeons often choose interventions based on clinical judgment or availability of surgical equipment. Bias in selection of participants at low to moderate risk (D2): most studies used consecutive patients with specific inclusion/exclusion criteria. However, many are single-center studies, which the authors acknowledge as a limitation. Bias in classification of interventions at low risk (D3): the surgical interventions (e.g., use of UO vs. HSB or laminectomy vs. fusion) were well documented. Bias due to deviations from intended interventions at moderate risk (D4): due to intraoperative adaptations. Bias due to missing data ranged from moderate to serious risk (D5): studies rated moderate had acceptable follow-up rates, whereas those rated serious experienced significant loss to follow-up without adequate statistical handling. Bias in measurement of outcomes at moderate risk (D6): the studies did not mention blinding of the assessors. Bias in selection of reported results at low risk (D7): most studies reported on all primary outcomes (neurological recovery and complications) consistent with their stated objectives.

Certainty of Evidence

Certainty of evidence evaluated using the GRADE approach is detailed in Table 4.

**Table 4:** Certainty of evidence for surgical management and prognosis of TOLF using GRADE mJOA = modified Japanese Orthopaedic Association Score; RR = recovery rate; CSF = cerebrospinal fluid; MRI = magnetic resonance imaging; SCR = signal change ratio on quantitative MRI; TOLF = thoracic ossification of the ligamentum flavum. ⨁◯◯◯ = very low; ⨁⨁◯◯ = low; ⨁⨁⨁◯ = moderate; ⨁⨁⨁⨁ = high. Certainty levels: High = very confident; Moderate = moderately confident; Low = limited confidence; Very low = very little confidence in the estimate. Downgrading rules: Observational studies started at “Low” certainty and were downgraded for serious concerns in risk of bias, inconsistency, indirectness, imprecision, or publication bias. Publication bias could not be formally tested due to absence of meta-analysis and small number of studies; therefore, it was judged qualitatively.

Outcome	No. of studies/No. of patients	Risk of bias	Inconsistency	Indirectness	Imprecision	Publication bias	Certainty of evidence	Importance
Neurological recovery (mJOA/RR)	10/846	Serious	Not serious	Not serious	Serious	Suspected	⨁◯◯◯ Very low	Critical
Complications (CSF leakage, dural tears, neurological deterioration)	10/846	Serious	Moderate	Not serious	Serious	Suspected	⨁◯◯◯ Very low	Critical
Prognostic factor: symptom duration (>12 to17 months)	5/379	Serious	Moderate	Not serious	Not serious	Suspected	⨁⨁◯◯ Low	Important
Prognostic factor: T2 hyperintensity/SCR on MRI	6/556	Serious	Moderate	Not serious	Not serious	Suspected	⨁⨁◯◯ Low	Important

Because all included studies were observational, the initial level of certainty was low. Several outcomes were downgraded further due to serious methodological limitations related to retrospective design and lack of control for confounding. Precision was also limited by small sample sizes and variability in outcome reporting. Publication bias could not be formally examined owing to the small number of studies and absence of quantitative synthesis, but was considered possible given the predominance of small, single-center reports. Consequently, evidence certainty was judged to be very low for neurological recovery and perioperative complications, and low for major prognostic factors including symptom duration and MRI signal characteristics.

Discussion

This systematic review identified several imaging and clinical factors that consistently influence surgical outcome in thoracic OLF. Symptom duration exceeding 12 months was strongly associated with poor surgical outcomes, likely due to irreversible damage such as demyelination [23]. A preoperative SCR ≥1.54 was an independent predictor of poor outcome. Thus, MRI plays an important prognostic role in addition to its diagnostic value, particularly through intramedullary T2 hyperintensity [20,23].

TOLF occurs most frequently in the lower thoracic spine (T10-T12), which is a transitional area subject to increased mechanical stress [19]. Du et al. performed a two-way analysis of variance (ANOVA), which demonstrated that while sagittal morphology significantly affected postoperative mJOA score and RR, the axial ossification had no such effect [25]. Beak-type sagittal morphology has been associated with greater compression and poorer neurological recovery in some studies [24,25], although this association has not been consistently demonstrated across cohorts [19].

The OLF morphology clearly influenced surgical technique selection, although its prognostic value remains debated. The bridged type (where bilateral ossifications meet in the midline) often requires a wider decompression area, increasing the risk of instability and the need for instrumentation and fusion [22,25]. Tuberous and round-type lesions have been associated with a greater incidence of intraoperative dural tears [27]. Beak-type ossification is often reported as being more difficult to remove, which contributes to a more challenging surgery and potentially less complete recovery. For patients with beak-type morphology, some authors suggest use of laminectomy with instrumentation rather than laminectomy alone [22]. 

Although not part of the formal SWiM synthesis domains extracted from our primary cohorts, a review of comorbidities and systemic factors provides important broader clinical context for these patients. Studies found that although diabetes did not independently influence final neurological outcome, it was associated with higher perioperative complication rates and greater patient dissatisfaction [19,26]. Diabetic patients experienced significantly higher rates of delayed wound healing (75%) versus the non-diabetics (26.7%) [26]. In contrast, comorbidities such as hypertension, cardiac disease, and cerebrovascular disease did not appear to significantly alter neurological outcomes [22,26,27]. Epidemiological evidence demonstrates a positive correlation between an elevated body mass index (BMI) and the prevalence of OLF, even among individuals without clinical symptoms [29]. This relationship indicates that obesity likely acts as a significant systemic driver in the pathogenesis and progressive development of heterotopic ossification within the spinal ligaments. Studies noted that severe obesity was more prevalent in patients with multilevel thoracic OLF, with overall severity of the ossified lesions compounding as BMI increases [30-33].

Limitations

This systematic review is primarily limited by the retrospective design of all included studies, which increases the risk of selection bias and confounding. Because symptomatic thoracic OLF is uncommon, most studies involved small patient cohorts, limiting statistical power and external validity. Furthermore, our synthesis relied heavily on vote counting based on statistical significance. This is a recognized methodological limitation as it does not account for effect size or precision, and is vulnerable to being swayed by underpowered studies failing to reach significance despite a true clinical effect. This was compounded by the fact that the reporting of statistical parameters varied significantly across the primary studies; many provided only p-values or raw percentages without the corresponding 95% confidence intervals, which limited the depth of our quantitative assessment. Follow-up duration was heterogeneous and often limited to approximately two years, which may not fully capture long-term neurological recovery. Outcome assessments, specifically mJOA scores and patient satisfaction, are inherently subjective and may be influenced by other factors like hospitalization expenses and pain perception.

Endoscopic and other minimally invasive surgical approaches were excluded because the review was designed to evaluate posterior decompression with or without instrumented fusion; therefore, the findings should not be extrapolated to these alternative techniques.

Additionally, the electronic search was limited to PubMed/MEDLINE and English-language publications, and relevant studies indexed exclusively in other databases may therefore have been missed. Furthermore, nine of the 10 included studies originated from China and one from India; although this reflects the recognized epidemiological predominance of TOLF in Asian populations, the geographic concentration of the available evidence may limit generalizability to other populations.

Finally, most studies were single-center series, with only two multicenter investigations identified, underscoring the need for future large-scale randomized controlled trials to provide more reliable evidence.

## Conclusions

Neurological and functional outcomes after surgery for TOLF are determined mainly by preoperative neurological status, duration of symptoms, and adverse MRI features rather than by the decompression technique alone. These variables should be utilized to guide patient selection, timing of intervention, and consideration of fusion. Adjunctive technologies such as UOs mainly influence perioperative safety without improving long-term neurological recovery. In view of the low certainty of current evidence, surgical decisions should remain individualized until higher-quality prospective data become available.
